# At-Risk Testing for Pompe Disease Using Dried Blood Spots: Lessons Learned for Newborn Screening

**DOI:** 10.3390/ijns6040096

**Published:** 2020-12-21

**Authors:** Zoltan Lukacs, Petra Oliva, Paulina Nieves Cobos, Jacob Scott, Thomas P. Mechtler, David C. Kasper

**Affiliations:** 1 Newborn Screening and Metabolic Diagnostics Unit, Hamburg University Medical Center, 20251 Hamburg, Germany; lukacs@uke.de (Z.L.); acquim@hotmail.com (P.N.C.); 2 ARCHIMED Life Science GmbH, 1110 Vienna, Austria; j.scott@archimedlife.com (J.S.); t.mechtler@archimedlife.com (T.P.M.); d.kasper@archimedlife.com (D.C.K.)

**Keywords:** newborn screening, Pompe disease, dried blood spots, Pompe disease diagnostics testing

## Abstract

Pompe disease (GSD II) is an autosomal recessive disorder caused by deficiency of the lysosomal enzyme acid-α-glucosidase (GAA, EC 3.2.1.20), leading to generalized accumulation of lysosomal glycogen especially in the heart, skeletal, and smooth muscle, and the nervous system. It is generally classified based on the age of onset as infantile (IOPD) presenting during the first year of life, and late onset (LOPD) when it presents afterwards. In our study, a cohort of 13,627 samples were tested between January 2017 and December 2018 for acid-α-glucosidase (GAA, EC 3.2.1.20) deficiency either by fluorometry or tandem mass spectrometry (MS). Testing was performed for patients who displayed conditions of unknown etiology, e.g., CK elevations or cardiomyopathy, in the case of infantile patients. On average 8% of samples showed activity below the reference range and were further assessed by another enzyme activity measurement or molecular genetic analysis. Pre-analytical conditions, like proper drying, greatly affect enzyme activity, and should be assessed with measurement of reference enzyme(s). In conclusion, at-risk testing can provide a good first step for the future introduction of newborn screening for Pompe disease. It yields immediate benefits for the patients regarding the availability and timeliness of the diagnosis. In addition, the laboratory can introduce the required methodology and gain insights in the evaluation of results in a lower throughput environment. Finally, awareness of such a rare condition is increased tremendously among local physicians which can aid in the introduction newborn screening.

## 1. Introduction

Pompe disease (GSD II) is an autosomal recessive disorder caused by deficiency of the lysosomal enzyme acid-α-glucosidase (GAA, EC 3.2.1.20), leading to a generalized accumulation of lysosomal glycogen especially in the heart, skeletal and smooth muscle, and the nervous system. It is generally classified based on the age of onset as infantile (IOPD) presenting during the first year of life, and late onset (LOPD) when it presents afterwards [1,2,3]. IOPD is usually associated with cardiomyopathy and then referred to as classic Pompe disease [1]. Infants with classic Pompe disease typically present during the first few weeks of life with hypotonia, progressive weakness, macroglossia, and hepatomegaly. Most of these infants die by their first birthday [4]. In the rare instances presenting without cardiomyopathy, it is referred to as non-classic Pompe disease [5,6,7].

The diagnosis of Pompe disease is usually made based on the typical clinical presentation followed by the demonstration of deficiency of GAA enzyme activity in muscle, skin fibroblasts or more recently dried blood spots (DBS) as well as GAA mutation analysis [2,8]. Diagnosis of Pompe disease through newborn screening (NBS) is also possible [9,10]. Prior to the initiation of enzyme replacement therapy, rapid determination of CRIM status [11,12] in patients with infantile onset Pompe disease is extremely important [13]. Depending on results immune-suppressive therapy may be initiated. Pompe disease is still considered to be a rare inborn error of metabolism with an estimated frequency of about 1/40,000 and a higher incidence in certain populations such as African Americans (1/14,000), Northern Europeans of Dutch origin and South East Asians [2]. Early results of newborn screening pilot studies from Taiwan [14] and the USA [15,16,17] indicated a higher general incidence, especially of LOPD cases which may be missed as clinical symptoms are less clear.

GAA catalyzes the hydrolysis of α1→4 glucosidic linkages in glycogen at acid pH [2]. Specificity for the natural substrate (glycogen) is gained during its maturation. The activity of mature (76/70-kDa) GAA for its natural (glycogen) substrate is considerably more robust than its activity towards the artificial substrate (4-methylumbelliferyl-α-d-glucoside; 4-MU), which is frequently used in in-vitro assays. However, 4-MU is also a substrate for several other enzymes including “leukocyte” neutral isoenzymes, glucosidase II (GANAB), neutral α-glucosidase C (GANC), and maltase glucoamylase (MGAM). Therefore, using maltose or, preferably, acarbose as an inhibitor of MGAM activity, allows for the measurement of GAA activity in DBS samples with minimal interference by other α-glucosidases. This assay serves as the basis for newborn screening and the non-invasive diagnosis of Pompe disease [18,19,20]. As a result, multiplex newborn screening assays for Pompe disease (based on GAA enzyme activity) and other lysosomal storage disorders using fluorometry, digital microfluidics or tandem mass spectrometry have been developed [10,21,22,23]. In addition for qualitative and quantitative assessments of the disease burden, and clinical measurement of the impact of Pompe disease on various affected systems, urinary glucose tetrasaccharide (Glc4), a biomarker of glycogen storage with 94% sensitivity and 84% specificity for Pompe disease, is frequently used in monitoring the response of patients to enzyme replacement therapy and as an adjunct to acid α-glucosidase activity measurements [24,25,26]. However, there is still no reliable biomarker to predict the natural course of the disease in an individual or the point of time when ERT should be administered for LOPD cases. This complicates the introduction of newborn screening in many areas. In contrast, diagnostic testing allows early detection of LOPD cases without ethical problems and may pave the way for a future introduction of whole population Pompe screening.

In this paper, we present the data from testing over 13,000 individuals suspected having of Pompe disease collected over a two-year period by two different centers in Europe (Hamburg, Germany and Vienna, Austria). At-risk testing is the use of the assay to determine whether an individual at increased risk of having Pompe disease (because they have family history or symptoms remotely associated with the disease but not pathognomic for the disease such as CK-elevations of unknown origin) has a deficiency of GAA. Further diagnostic testing will be required for individuals whose test is suggestive of the condition to confirm true deficiency of enzyme or for example a compromised sample, as sample quality and shipping conditions can affect the results.

## 2. Materials and Methods

A DBS kit containing a customized card (Whatman 903) for blood sampling and sampling instructions, and an envelope was provided to physicians upon request for α-glucosidase testing. Dried blood specimens were received with brief clinical details and an ICF (informed consent) between January 2017 through December 2018. DBS protocols used to measure α-glucosidase (EC:3.2.1.20) enzyme activities are given below.

### 2.1. Fluorometric Method

The method by Chamoles et al. [18] was slightly modified. A 3 mm DBS was eluted with 360 μL of demineralized water, then 40 μL aliquots transferred to a 96-well plate. The test was run using the artificial substrate 4-methylumbelliferyl-α-d-glucoside (1.4 mM, Sigma-Aldrich, Darmstadt, Germany) in 40 mM sodium acetate (CH3COONa) buffer at pH 3.8 (Merck, Darmstadt, Germany) with and without the addition of 10 μL of 80 μM acarbose solution (Toronto Research Chemicals, North York, ON, Canada). The assay was also performed at pH 7.0 (40 mM CH3COONa buffer adjusted with hydrochloric acid/sodium hydroxide (HCl/NaOH) to pH 7.0), to assess the quality of the DBS. The α-glucosidase activity at pH 7.0 is a convenient tool for quality assessment because the same buffers adjusted to pH 7.0 can be used as for the target enzyme at pH 3.8. In addition, the enzyme activity at pH 7.0 is usually less stable than α-glucosidase activity at pH 3.8 when subjected to detrimental pre-analytical conditions, thereby being an early marker for specimen quality. All tests were run in duplicate. After incubation for 21 h at 37 °C, the reaction was stopped by the addition of 200 μL EDTA buffer (150 μM, pH 11.5; Sigma-Aldrich). The 40 μL of DBS eluate that had been stored at 4 °C overnight was added to specific wells that served as blanks. A standard curve of 4-methylumbelliferone (0 to 3 μM (Sigma-Aldrich)) run on each plate was used for the calculation of enzyme activities. The fluorescence was read on a Victor D instrument (Wallac Oy, Turku, Finland) or a BioTek Synergy H1 (Bad Friedrichshall, Germany). In addition to enzyme activity, the percent inhibition with acarbose and the ratio of α-glucosidase activities at pH 3.8 with inhibition to the activity at pH 7.0 were calculated to aid in the interpretation of results [27]. For samples from patients older than 1 year of age, a truncated assay which relied only on the measurement of α-glucosidase with acarbose inhibition was used. As a reference enzyme, β-galactosidase was run on these samples. Specimens from infants and those that showed diminished α-glucosidase activity in the truncated test, were analyzed using the test with and without acarbose. The calculation of additional ratios allowed for a better interpretation of results from samples with borderline values. Individuals from whom specimens with normal results in the truncated assay were considered not affected by Pompe disease and this assay allowed higher throughput testing and expedited reporting of results.

### 2.2. MS Method

The assay was based on previously published methods [9,28]. The samples were processed using the following steps. The activities of acid β-glucocerebrosidase (ABG; Gaucher disease), acid sphingomyelinase (ASM; Niemann-Pick A/B disease), α-glucosidase (GAA; Pompe disease) and α-galactosidase; GLA; Fabry disease) were measured in a multiplex assay. The extract from one 3.2 mm punch per DBS sample was combined with substrate and internal standard (S&IS) mixtures. Incubation was performed at 37 °C for 20–22 h. The reaction was stopped by adding 100 µL stopping solution (80% acetonitrile plus 0.2% formic acid in water). Aliquots were transferred to a new deep-well plate, covered with aluminum foil and centrifuged at 3000× *g* for 15 min prior to mass spectrometry analysis. Background activity of a blank blood collection paper spot was subtracted from the DBS activity. Two QC samples with previously established activity levels for each enzyme and heat inactivated samples were included in each plate as assay controls.

## 3. Results

Between January 2017 and December 2018 13,627 specimens were tested for GAA deficiency by fluorometry or tandem mass spectrometry (MS) using Dried Blood Spots (DBS) (Table 1). Specimens came from 51 different countries but most were from Germany, Poland, Turkey, Italy and Iran. Approximately 30% of all samples submitted were from infants. The median age was 17 years and the range 1–95 years. Specimens from individuals with family history of the condition were excluded.

Most of the tested samples (92%) showed normal enzyme activity. 8% of the samples showed enzyme activity below the respective cut-off. In most cases with decreased enzyme activity (419 from the fluorometric method and 696 from the MS method) genetic analysis was performed on the same bloodspot. 35–40% of the low enzyme samples screened by the MS method were genetically confirmed with two pathogenic variants, and similar confirmation rates have been obtained for the fluorometric method. Fluorometry has lower sensitivity in comparison to mass spectrometry [29].

Some positive results were obtained in specimens affected by detrimental pre-analytical conditions which did not necessarily reduce other enzyme activities. In those instances, a second card was requested for analysis.

Unfortunately, not all DBS came with a description of clinical symptoms. However, the major symptoms that were given are summarized in Table 2 grouped by analogous symptoms and sorted by severity. Cardiomyopathy was present almost exclusively in infant cases while CK-elevations of unknown origin or limb girdle muscle dystrophy of unknown origin were more prominent among LOPD cases in the Hamburg cohort.

Statistical results of the GAA enzyme activity measurement using either fluorimetry or tandem mass spectrometry are listed in Table 3.

The effect of drying conditions on GAA enzyme activity was studied. Duplicate specimens were collected. One was dried at room temperature overnight (dry) while the other remained in a plastic wrapping for 2 days to simulate transport without proper drying (wrapped). After this time period the sample was taken out of the plastic bag and dried overnight. Both were tested using the fluorometric method with and without addition of acarbose and as at pH 7.0 (reference enzyme). A significant decrease of enzyme activity (on average 50%) was observed if samples were not dried properly. In two cases (sample 2 and 3) it led to results that could be interpreted as consistent with Pompe disease or carrier status, while the interpretation of sample 5 changed from borderline positive into an unsuitable specimen. This observation is consistent with previously reported results [30]. Data are summarized in Table 4.

## 4. Discussion

Newborn screening for Pompe disease can be performed by a variety of methods based on enzyme activity measurement. NBS provides early identification of both classic severe IOPD and less-severe LOPD patients. With early detection and ERT, there are benefits for classic severe IOPD patients, however current therapy has limitations, especially with respect to the neurologic manifestations of the disease. The combination of early detection, close monitoring, and early ERT may be beneficial to less-severe LOPD patients as well. However, in some countries and regions the identification of LOPD presents an ethical problem and whole population screening poses financial constraints on health care systems. In contrast, at-risk testing as presented here, may be a potential first step. It allows for earlier identification of IOPD cases and potentially also LOPD when performed with a targeted approach using nonspecific symptoms loosely associated with Pompe disease, such as CK elevations of unknown origin. Interestingly, usually mild to moderate CK elevations are observed in patients with Pompe disease, which do not improve under therapy. In our study we have received specimens from patients who presented with non-specific symptoms and thus, may have Pompe disease but may have had another disorder hence GAA measurement aids in the differential diagnosis. Among 13,627 samples tested, 8% had decreased enzyme activity. We have shown that detrimental pre-analytical conditions may also affect enzyme activities negatively, so further confirmation is necessary. For that purpose, another enzyme measurement can be performed, and a molecular genetic assay should also be carried out. Previously, we have demonstrated that about. 2% of patients tested are eventually confirmed with Pompe disease which is in agreement with other international studies [31,32]. This demonstrates the high efficacy of the at-risk testing approach. The time to diagnosis can be significantly shortened, especially for LOPD patients who present with less specific symptoms. For IOPD patients, testing may be helpful in regions that are not familiar with the specific symptoms however due to the first clear symptoms, in particular floppiness, which occurs around 2–3 months of age, an even earlier diagnosis remains restricted to newborn screening.

Interest in Pompe disease testing within NBS programs has increased substantially in recent years. Sample quality greatly affects results from Pompe testing and newborn screening in general. As previously described [30], the combination of humidity and heat has the greatest impact on enzyme stability. The authors also showed with their shipping study that when properly dried DBS were stored in either paper envelopes or plastic bags, enzyme activities remained essentially intact for nine days in the US postal setting [30]. In contrast, insufficient drying combined with shipping of the samples in sealed (plastic) containers leads to grossly reduced activity, especially of the acid α-glucosidase and may even result in erroneous interpretation of the results as the activity of the reference enzyme remains in the normal range.

For adequate samples, GAA levels in specimens from affected patients are well resolved from those observed in specimens from healthy subjects using either fluorometry or tandem mass spectrometry (Table 3). The strength of mass spectrometry lies especially in its ability to measure several enzyme activities simultaneously. This is beneficial for newborn screening when various different lysosomal storage disorders are included in a national panel. Furthermore, the different enzyme activities may aid in the evaluation of sample quality and thus differentiate the cause of low enzyme activity between deficiency in the individual and deficiency caused by inappropriate storage conditions or transport. Using the fluorometric method as described the activity of α-glucosidase at pH 7.0 can be measured. This is usually less stable than the acid α-glucosidase and therefore, is a good indicator of negative environmental influences. For α-glucosidase tests which only include the activity with inhibition, β-galactosidase may be an alternative reference enzyme to monitor sample quality. This additional fluorometric test is fast and inexpensive, however, it must be used with some caution. β-galactosidase in dried blood may be relatively stable, therefore, a low enzyme activity result for α-glucosidase may still be caused by pre-analytical conditions rather than an actual disease. This applies to reference enzymes in general, as different environmental conditions may affect these enzymes to varying degrees. Thus, despite problematic conditions prior to the arrival of the specimen in the laboratory, the reference enzyme(s) may still show normal activity levels in the DBS. Therefore, the assessment of another specimen, either again in DBS or in a different material should always be considered as a second step. For further confirmation, a molecular genetic assessment is necessary. In case of IOPD, it may replace the second enzyme assessment or can be carried out in parallel to save valuable time and initiate therapy more rapidly.

In conclusion, at-risk testing can provide a good first step for the future introduction of Pompe disease to a newborn screening program as the laboratory can introduce the required methodology and gain insights in the evaluation of results in a lower throughput environment. It yields immediate benefits for the patients regarding availability and timeliness of the diagnosis. Finally, awareness of this rare condition is increased tremendously among local physicians which can aid in the introduction of Pompe disease into a national newborn screening program.

## Figures and Tables

**Table 1:** Number of samples tested between January 2017 and December 2018 at the specialized centers in Hamburg, Germany and Vienna, Austria.

	Fluorometry Method	MS Method
Number of tests	7340	6287
Normal enzyme activity	6921	5591
Enzyme activity below cut-off (positive)	419 (6%)	696 (11%)

**Table 2:** Clinical symptoms provided for samples submitted to the study centers in Hamburg and Vienna. Not all specimens contained such information.

	Clinical Presentation
1	Cardiomyopathy
2	Hypotonia—floppy baby, proximal and progressive muscle weakness, limb girdle muscle weakness, muscle pain, loss of strength and myalgia
3	Scoliosis myopathy, rigid spine, diffuse myopathy, myopathic syndrome, EMG: myogenic involvement, motor deficit of the belt
4	Elevated biomarkers—CK, myoglobin, transaminases

**Table 3:** Statistical results for both reference methods used in Hamburg (fluorometry) and Vienna (MS). For the normal values all specimens from individuals considered unaffected by Pompe disease have been included.

	Fluorometry Method [µmol/punch/h]		Tandem Mass Spectrometry Method [µmol/L/h] with Acarbose
	α-Glucosidase with Acarbose	α-Glucosidase without Acarbose	
Mean	7.43 × 10^−8^	1.24 × 10^−7^	9.24
Median	6.57 × 10^−8^	1.05 × 10^−7^	8.12
1st Percentile	2.14 × 10^−8^	5.05 × 10^−8^	4.69
99th Percentile	2.28 × 10^−7^	3.75 × 10^−7^	
99.9th Percentile	3.84 × 10^−7^	5.42 × 10^−7^	
Reference range	4.29–34.29 × 10^−8^	7.14–47.62 × 10^−8^	
Affected range	<0.4	n/a	3.3

**Table 4:** Degradation study of GAA activities for five dried blood specimens that showed different index activities. Samples were either dried overnight after spotting (dry) or put into sealed plastic bags immediately, in order to simulate shipping without proper drying (wrapped). After 2 days the samples were taken out and allowed to dry overnight. Both specimens were tested in the same run in duplicates. All samples showed a significant decrease in activity when the specimen was not dried before shipping.

α-Glucosidase Activity [nmol/punch × 21 h] (Reference Range)	Sample 1		Sample 2		Sample 3		Sample 4		Sample 5	
	Wrapped	Dry	Wrapped	Dry	Wrapped	Dry	Wrapped	Dry	Wrapped	Dry
pH 3.8	2.07	3.02	0.82	2.06	1.28	3.09	1.92	2.56	0.29	1.6
(>1.5)										
pH 7.0	5.4	6.47	1.8	4.48	3.33	6.12	4.07	6.04	0.9	5.52
(>1.8)										
pH 3.8 with acarbose	1	1.72	0.54	1.19	0.72	1.86	1.12	1.35	0.18	0.58
(>0.9)										
